# Green and resilient hotel operations through mega-event legacies

**DOI:** 10.3389/fspor.2025.1604131

**Published:** 2025-08-15

**Authors:** Toyirova Sarvinoz Atoevna, Lourdes Canós-Darós, Estefanía Osorio-Acosta

**Affiliations:** ^1^ Department of Business Organization, Universitat Politècnica de València, Valencia, Spain; ^2^ Tourism and Hotel Management Department, Faculty of Economics and Tourism, Bukhara State University, Bukhara, Uzbekistan

**Keywords:** mega-events, sustainable tourism, green hotel operations, resilience, event legacy, hospitality management, community impact

## Abstract

Mega-events such as the Olympic Games, World Cup, and World Expo aim to leave sustainability legacies, yet the mechanisms through which these goals are realized in hotel operations remain underexplored. This study adopts a multiple case study design, analyzing secondary data including sustainability reports, certification documents, and post-event assessments from five host cities—London, Tokyo, Dubai, Rio de Janeiro, and Doha. The findings reveal that mega-events can serve as catalysts for sustainability upgrades in hotels, including energy-efficient technologies, employee training, and guest-oriented green initiatives. However, not all legacies are uniformly positive: issues such as short-lived initiatives, cost burdens, and limited accountability mechanisms persist. This study contributes to tourism and event management literature by demonstrating how hotels, as essential components of tourism infrastructure, operationalize event-time sustainability commitments into longer-term environmental and social value. It also highlights the interplay of motivations (branding, regulation, guest expectations), constraints (financial and operational), and outcomes (emissions reduction, organizational resilience). While many upgrades offer both financial and reputational benefits, others require trade-offs, confirming that sustainability legacies are complex, context-dependent, and not universally “win-win”.

## Introduction

Mega-events such as the Olympic Games, World Cup, and World Expo aim to leave sustainability legacies (94, 95), yet the mechanisms through which these goals are realized in hotel operations remain underexplored. This study adopts a multiple case study design, analyzing secondary data including sustainability reports, certification documents, and post-event assessments from five host cities—London, Tokyo, Dubai, Rio de Janeiro, and Doha. The findings reveal that mega-events can serve as catalysts for sustainability upgrades in hotels, including energy-efficient technologies, employee training, and guest-oriented green initiatives. However, not all legacies are uniformly positive: issues such as short-lived initiatives, cost burdens, and limited accountability mechanisms persist. This study contributes to tourism and event management literature by demonstrating how hotels, as essential components of tourism infrastructure, operationalize event-time sustainability commitments into longer-term environmental and social value. It also highlights the interplay of motivations (branding, regulation, guest expectations), constraints (financial and operational), and outcomes (emissions reduction, organizational resilience). While many upgrades offer both financial and reputational benefits, others require trade-offs, confirming that sustainability legacies are complex, context-dependent, and not universally “win-win”.

Central to all these attempts at sustainability are hotels, which are the major venues for accommodating the sheer volume of visitors flooding in during mega-events. Hotels are not only among the largest components of a mega-event's environmental footprint through energy, water use, and waste generation (1, 2), but they can also perform pretty well in providing green practices to a global audience and demonstrating the viability of sustainable hospitality operations on a mass scale. The hospitality sector's contribution to mega-event sustainability goes beyond operational efficiency to encompass workforce development, community engagement, and building long-term resilience in destinations.

A driving force behind the push for sustainability is the increasing public expectation for environmental responsibility. Global travelers are more eco-conscious than ever: the World Tourism Organization (UNWTO) reported in 2023 that approximately 73% of tourists prefer to stay in hotels that implement sustainable practices (3). This consumer pattern positions hospitality providers at the leading edge of event sustainability efforts. Hotels accommodate the influx of visitors for mega-events and thus are a major contributor to the event's environmental footprint in energy use, water consumption, and waste generation (1, 2). Hotels additionally have the unique potential to showcase green practices to an international audience and capture the gains of the growing niche of environmentally concerned tourists. The majority of hotel companies have responded by adopting sustainability certifications (e.g., Green Key, EarthCheck, or LEED) and green practices to meet both governmental requirements and traveler expectations (4, 5). For instance, Marriott International has pledged that 100% of its hotels will be certified to an internationally recognized sustainability standard by 2025 (6)—an ambitious goal reflective of industry-wide momentum.

However, it is crucial to acknowledge that not all sustainability efforts provide direct financial returns. While eco-efficiency measures such as energy and water conservation often yield measurable cost savings through reduced utility bills, other important sustainability initiatives may offer primarily indirect or long-term value (7, 8). For example, investments in workforce well-being, community engagement programs, biodiversity conservation, or renewable energy infrastructure may not show immediate return on investment (ROI) but contribute to social sustainability, brand reputation, and long-term resilience (9, 10). This complexity is particularly relevant in the context of mega-events, where hotels may be required to implement comprehensive sustainability measures that go beyond those with clear financial benefits. Understanding this nuanced relationship between sustainability initiatives and their varied outcomes—financial, environmental, and social—is essential for both practitioners and researchers examining the legacy of mega-events on hotel operations.

## Literature review

The concept of legacy in mega-events has evolved significantly over the past two decades. Early conceptualizations focused primarily on economic and infrastructural outcomes (11, 12), but contemporary understanding encompasses environmental, social, and cultural dimensions (13, 14). Preuss (15) refined his earlier definition to emphasize that legacies can be both tangible (infrastructure, venues) and intangible (knowledge transfer, social capital), and that they may manifest differently across stakeholder groups.

For hotels, mega-event legacies are felt in terms of shifts in operations, infrastructure upgrading, and capacity building that lasts beyond the event horizon. These legacies may be comprised of physical capital (energy systems, waste management plants), human capital (skilled staff, sustainability experience), and organizational capabilities (environmental management systems, stakeholder engagement processes). However, theoretical concepts of how these legacies for hotels are built, maintained, and measured are still lacking. This study contributes to legacy theory by investigating the specific processes through which hotels translate event-time sustainability pressures into sustainable operational changes.

Environmental sustainability in mega-events gained prominence following criticism of the ecological impacts of earlier events (16, 17). The Sydney 2000 Olympics marked a turning point with its “Green Games” agenda, though scholars have debated the extent to which these aspirations were realized (18, 19). Subsequent events have increasingly incorporated sustainability into bid documents and operational planning, driven by both regulatory requirements and reputational concerns (20).

The hospitality industry's engagement with sustainability has been extensively documented in academic literature. Early work by Kirk (21) and Bohdanowicz (22) established the business case for environmental management in hotels, focusing primarily on eco-efficiency measures. More recent scholarship has expanded to examine social sustainability dimensions, including workforce development, community engagement, and cultural preservation (23, 24).

Institutional theory provides an effective framework for analyzing how mega-events influence hotel sustainability adoption. DiMaggio and Powell (25) identified three sources of institutional pressures for organizational change: coercive (regulations, mandates), normative (professional standards, certifications), and mimetic (imitation of successful practices). Mega-events create all three sources of pressure simultaneously because hotels are exposed to regulatory demands, industry norms, and competitive pressure to implement sustainable practices. However, the between-event and between-setting heterogeneity of results suggests that institutional pressures alone are not enough to guarantee implementation success—organizational capacity and commitment remain necessary (26). The concept of resilience in hotel operations has gained increasing attention, particularly following shocks such as the COVID-19 pandemic. Resilience can be conceived as both the ability to cushion shocks and the ability to learn and transform with changing conditions (98, 99). For hotels, sustainability initiatives undertaken during mega-events can contribute to operational resilience through reducing resource dependencies, diversifying revenues, and building adaptive capacity. This study contributes to resilience theory by examining how mega-event sustainability initiatives enhance hotels' adaptive capacity and long-term viability. The analysis reveals that hotels that successfully implement comprehensive sustainability programs during mega-events often develop organizational capabilities that enable them to respond more effectively to future challenges and opportunities.

Certification systems have emerged as key mechanisms for standardizing and communicating sustainability practices in hospitality (27, 28). Industry-specific certifications like LEED have seen increasing adoption in the hospitality sector, with over 1,000 hotels globally achieving certification by 2021 (73). However, research has identified several challenges, including the proliferation of competing standards (29), varying levels of rigor (30), and questions about their effectiveness in driving genuine change vs. “greenwashing” (31). Studies by Geerts (32) and Pereira et al. (33) found that while certifications can drive operational improvements, their impact on actual environmental performance varies considerably based on implementation quality and management commitment. A critical consideration in evaluating sustainability initiatives is the well-documented gap between stated consumer preferences and actual behavior. While surveys consistently show high levels of consumer interest in sustainable tourism options, actual purchasing decisions often prioritize price and convenience (34, 35). This phenomenon, known as the intention-behavior gap or attitude-behavior gap, has significant implications for hotels investing in sustainability measures based on guest preference surveys (36, 37). The business case for hotel sustainability is further complicated by the reality that not all sustainability initiatives provide direct financial returns. While eco-efficiency measures often demonstrate clear ROI, comprehensive sustainability requires acceptance of initiatives without immediate financial benefits (8). These challenges simplistic “win-win” narratives and aligns with critical perspectives on corporate sustainability that emphasize trade-offs and competing objectives (38, 39). Research has identified various factors contributing to this gap, including:

• Price sensitivity and perceived value trade-offs (40)
• Lack of transparent information about sustainability practices (41)
• Habitual behaviors that override intentions (42)
• Social desirability bias in survey responses (43)

## Methodology

This study employs a multiple case study approach, which is particularly suited for examining complex phenomena in real-world contexts where boundaries between phenomenon and context are not clearly defined (44). The case study methodology has been widely used in tourism and hospitality research to investigate sustainability initiatives (45, 46) and mega-event legacies (47, 48).

Following Eisenhardt's (49) recommendations for theoretical sampling in case study research, we selected cases that would provide both literal replication (similar results for predictable reasons) and theoretical replication (contrasting results for predictable reasons). The selection criteria included:

1. Temporal diversity: Events spanning from 2012–2022 to capture evolving sustainability practices
2. Geographic distribution: Different continents and development contexts
3. Event types: Both sporting (Olympics, World Cup) and non-sporting (Expo) mega-events
4. Documented sustainability commitments: All selected events made explicit sustainability claims
5. Data availability: Sufficient documentation for analysis

The selected cases are:

• Summer Olympic Games [London 2012 (50), Rio de Janeiro 2016, Tokyo 2020]
• FIFA World Cup 2022 (51) (Qatar)
• World Exposition 2020 (Dubai)

This selection aligns with Stake's (52) collective case study approach, where multiple cases are examined to investigate a phenomenon, population, or general condition.

Data were collected from multiple sources to enable triangulation and enhance validity (53, 54):

1. Official documentation: Sustainability reports, legacy plans, environmental impact assessments
2. Academic literature: Peer-reviewed articles analyzing specific events
3. Industry reports: Hotel certification databases, trade publications, corporate sustainability reports
4. News media: Contemporary coverage and post-event analyses from reputable sources
5. NGO assessments: Independent evaluations from environmental and human rights organizations

## Data analysis

The analysis followed Braun and Clarke's (55) thematic analysis framework, adapted for case study research:

1. Familiarization: Immersion in data through repeated reading
2. Initial coding: Generation of initial codes for sustainability initiatives and outcomes
3. Theme development: Grouping codes into potential themes
4. Theme review: Checking themes against coded extracts and entire dataset
5. Theme definition: Refining and naming themes
6. Cross-case synthesis: Identifying patterns across cases (56)

The coding framework encompassed:

• Sustainability initiatives: Energy, water, waste, transportation, social programs
• Implementation mechanisms: Certifications, regulations, partnerships
• Outcomes: Environmental, economic, social, legacy dimensions
• Challenges: Financial constraints, operational difficulties, stakeholder conflicts

This study relies on secondary data, which presents several limitations:

• Potential bias in official reports toward positive outcomes
• Varying levels of detail and transparency across sources
• Limited access to internal hotel operational data
• Temporal constraints in assessing long-term legacies

We addressed these limitations through source triangulation and explicit acknowledgment of conflicting evidence where it exists.

## Results

Past mega-events have implemented various sustainability measures with mixed success. Table 1 provides a comparative analysis of significant sustainability measures and their outcomes.

**Table 1:** Sustainability initiatives and outcomes in recent mega-events.

Mega-Event	Key Sustainability Initiatives	Highlights and Outcomes	Sources
London 2012 Olympics	- Built Olympic Park on remediated brownfield land- Designed venues with sustainable materials- First Olympics to measure total carbon footprint- “Zero waste to landfill” commitment- Emphasized public transport	*Largely successful:* Achieved ∼400,000 tons CO₂ savings (28% reduction). Met most targets. However, energy management during events was criticized by watchdog reports.	UNEP (57); Commission for a Sustainable London 2012 (58); GHD (59)
Rio 2016 Olympics	- Comprehensive legacy plan including Guanabara Bay cleanup- “Green Passport” program- Urban transport upgrades (metro, BRT)	*Mixed results:* Transport infrastructure delivered lasting benefits. Environmental cleanup goals largely unmet (only 50% of bay cleanup achieved). Political instability affected implementation.	UNEP (60); Goldenbaum & Galante (2021); Rio 2016 Official Report (61)
Tokyo 2020 Olympics	- Pursued “carbon-neutral” Games- 100% renewable electricity- Recycled medals from e-waste- Hydrogen-powered facilities	*Innovation-focused:* Successfully demonstrated circular economy principles. Medals made from 78,985 tons recycled electronics. Limited spectator presence due to COVID-19 reduced some impacts.	Tokyo 2020 Sustainability Report (62); Teo (63)
Expo 2020 Dubai	- Sustainability as core theme- Terra Pavilion (net-zero building)- 130+ LEED certified buildings- 85% waste diversion rate	*Comprehensive approach:* Created lasting sustainable infrastructure. Site transformed into Expo City Dubai for continued use. Strong integration of sustainability throughout event.	Expo 2020 Sustainability Report (64); Gulf News (65)
Qatar 2022 World Cup	- Stadium 974 (dismantlable venue)- “Carbon-neutral” claim- New public transport system- Required hotel certifications	*Controversial:* Infrastructure improvements noted, but carbon-neutral claims widely disputed. Human rights concerns, including forced labor issues, overshadowed sustainability narrative.	FIFA (66); Boykoff (67); Human Rights Watch (68)

**Table 2:** Major hotel sustainability certifications.

Certification	Focus and Scope	Adoption Patterns	Evidence of Mega-Event Influence	Sources
Green Key	Operational sustainability across 13 categories	7,500+ establishments in 80+ countries	UEFA Euro 2016 required all official hotels to obtain certification. Some evidence of accelerated adoption in host cities pre-event.	FEE (69); Green Key Global (70)
EarthCheck	Science-based benchmarking and certification	550+ certified properties globally	Official partner for 2018 Gold Coast Commonwealth Games. Several Doha hotels pursued certification for Qatar 2022.	EarthCheck (71); Swindells (72)
LEED	Green building design and construction	1,000+ hotels globally	New hotel construction for events often pursues LEED. Tokyo saw increase in applications 2018–2020.	USGBC (73); Green Lodging News (74)
ISO 14001	Environmental management systems	Widely adopted by chains	Beijing 2008 and Tokyo 2020 official hotels encouraged to obtain. Difficult to isolate event impact from general trends.	ISO (75); Various corporate reports

The proliferation of sustainability certifications in the hospitality sector reflects growing industry engagement with environmental and social responsibility (Table 2). However, evidence of direct causal links between mega-events and certification adoption requires careful examination.

Hotels implement a variety of sustainability practices for mega-events, though the extent to which these are event-driven vs. reflecting broader industry trends must be closely analyzed. The evidence suggests that mega-events serve as accelerators of pre-existing sustainability trends rather than creating entirely new practices.

Energy conservation is the most common mega-event sustainability practice among hotels, driven by environmental as well as cost-saving imperatives. London 2012 hotels attained 15%–20% energy savings by implementing LED retrofits, smart building systems, and renewable energy sourcing. Tokyo 2020 featured more advanced measures, with AI-driven energy management systems providing additional 10% savings in some properties. These advances, however, reflect broader industry trends toward energy efficiency that have been accelerated by demands and visibility created by the events.

The longevity of energy conservation actions after the event differs significantly. Those hotels that incorporated energy management into mainstream operations sustained gains, whereas those that took temporary actions tended to revert to former routines. This trend indicates that durable energy efficiency legacies depend on systematic organizational transformations instead of discrete technological initiatives.

Water conservation measures for mega-events build on those already practiced, such as linen reuse programs and low-flow fixtures, in hotels. Over 90% of London's hotels had reuse programs by 2012, though the extent to which this was event-driven adoption vs. business-as-usual practice is unclear. More advanced water conservation measures, such as greywater reuse and leak detection systems, were more obviously event-demand driven.

Hilton Tokyo Bay's 5,000-liter saving during the Games shows how events can trigger intensified monitoring and optimization of existing systems. But the relatively limited scale of these gains suggests that water conservation for mega-events is a question of intensification of normal practice rather than transformational operational change.

Mega-event waste management improvements usually highlight the most creative and long-lasting transformation of hotel operations. FIFA partnerships on waste targets led to system-wide solutions that extended beyond individual properties. Marriott's elimination of plastic straws by 2019 (76), while part of an overall corporate plan, was accelerated because of mega-event sustainability pressures.

Expo 2020 hotels' contribution of 50,000 meals reflects wider incorporation of circular economy principles that have the potential to establish long-term operational transformation. Such practices tend to continue after the event due to alignment with both sustainability objectives and operational efficiency, generating reinforcing incentives for perpetuation.

Sustainable F&B at mega-events typically prioritize local supply, vegetarianism, and waste reduction. London 2012's Food for Life certification and Paris 2024's 50% local supply commitment demonstrate how events can formalize and accelerate existing trends for sustainable hospitality practices.

The integration of sustainable F&B practices has a tendency to extend beyond the event timeline as they can help optimize guest experience and reduce costs. Hotels that develop local supply chain connections and sustainable menu options during events are likely to sustain these efforts as they become engrained in operational routines and guest expectations.

One of the most significant long-term mega-event sustainability initiative legacies in hotels is employee training programs. Beijing (77) city-wide employee training programs created long-lasting human capital that persisted well beyond the event. These initiatives have a tendency to develop organizational competencies that enable hotels to achieve sustainability performance gains by themselves well beyond the event.

However, retention of sustainability knowledge and practices is dependent to a large degree on staff turnover rates and ongoing organizational support. Hotels with formal knowledge management processes and ongoing training exhibited greater sustainability improvement persistence instigated by events.

The mega-event legacies for hotel infrastructure and destination sustainability are extremely diverse, reflecting differences in planning, implementation, and post-event management. These legacies are of particular interest to hotel operations in that they establish the context within which individual properties develop their sustainability plans. The long-term impacts of mega-events on host destinations vary considerably, reflecting differences in planning, implementation, and post-event management.

Note: The long-term impacts of mega-events on host destinations vary considerably, reflecting differences in planning, implementation, and post-event management. To enhance clarity and enable more detailed analysis, we present these findings in two complementary tables: Table 3a examines infrastructure legacies across the five cases, while Table 3b specifically analyzes the implications for hotel operations and sustainability outcomes.

**Table 3a:** Infrastructure *legacy from mega-events.*

Event	Infrastructure Legacy	Long-term Sustainability Assessment	Sources
London 2012	- Olympic Park transformed to Queen Elizabeth Park - Athletes' Village → 2,800 housing units - Permanent transport improvements	Strong long-term sustainability trajectory. Infrastructure legacy supported continued green tourism development.	Visit Britain (78); UK Government (79); GHD (59)
Rio 2016	- 160 km BRT lines (lasting benefit) - Incomplete metro extensions - Some venues abandoned	Limited long-term sustainability legacy. Political and economic instability undermined systematic post-event sustainability development.	Al Jazeera (80); Rio Tourism Board (81)
Tokyo 2020	- Venues adapted for community use - Hydrogen infrastructure development - Accessibility improvements	Strong innovation legacy with potential for long-term impact. Technology development and accessibility standards continue to benefit sector.	JNTO (82); Tokyo Metropolitan Government (83)
Expo 2020 Dubai	- Site conversion to Expo City - Permanent metro extension - Innovation infrastructure	Excellent long-term prospects due to permanent infrastructure and continued use. Strong foundation for continued sustainable development.	Dubai Tourism (84); EY (85)
Qatar 2022	- $200B infrastructure investment - New metro system - Stadium legacy plans varied	Mixed legacy with strong infrastructure but concerns about ongoing sustainability commitment. Long-term impact uncertain.	Qatar Tourism (86); Various analysts

**Table 3b:** Hotel sector implications and operational impacts.

Event	Hotel Sector Impact	Operational Benefits	Sustainability Implications for Hotels
London 2012	Enhanced destination sustainability credentials. Continued tourism growth supported hotel investments in green infrastructure.	Transport improvements reduced guest carbon footprints. Hotels maintained and expanded event-period improvements.	Strong integration of sustainability practices into standard operations. Lasting employee capabilities and systems.
Rio 2016	Mixed impact on hotel sector sustainability. Economic challenges limited continued sustainability investments.	Transport improvements benefited some properties more than others. Limited operational transformation.	Hotel improvements largely temporary. Lack of systematic approach prevented lasting operational changes.
Tokyo 2020	Technology showcase benefits for hotel innovation. Limited immediate impact due to COVID-19 restrictions.	Accessibility improvements created lasting infrastructure advantages. AI and automation advances.	Hotel sector positioned to benefit from continued technology development and enhanced accessibility standards.
Expo 2020 Dubai	Comprehensive sustainable infrastructure creation. Permanent hotel facilities with integrated sustainability systems.	Integrated sustainable systems reduce operational costs. Innovation focus drives continuous improvement.	Hotel sector benefits from permanent infrastructure and ongoing innovation ecosystem. Strong foundation for leadership.
Qatar 2022	Significant infrastructure improvements benefiting hotel operations. Reputational challenges persist.	New transport systems improve guest access. Sustainability infrastructure investments support operations.	Hotel sector benefits from infrastructure but faces continued scrutiny on sustainability practices and commitments.

## Discussion

This study contributes theoretically by building on Preuss's (15, 87, 88) legacy frameworks and the adaptive systems literature. Specifically, it examines how event-time sustainability efforts catalyze longer-term adaptive capacity in hotels, thus extending resilience theory. The empirical findings challenge and enrich these frameworks by emphasizing the importance of sector-specific implementation mechanisms and organizational routines.

Our findings contribute to several theoretical discussions in tourism and event management literature. First, we extend legacy theory by demonstrating how sustainability initiatives create multi-dimensional impacts that transcend traditional economic and infrastructural outcomes (88). The cases reveal that environmental legacies are deeply intertwined with social and economic dimensions, supporting integrated approaches to legacy planning (13, 89).

Specifically, this study advances legacy theory by identifying the mechanisms by which hotels translate transient event pressures into resilient organizational capabilities. The results suggest that successful legacy creation is not only about initial adoption of sustainable practices, but also creating organizational routines, systems of knowledge, and stakeholder relations that contribute to continuous improvement. This result extends Preuss's (15) legacy conceptualization to emphasize the importance of adaptive capacity in predicting whether event-induced changes are sustained and evolve.

Second, our analysis provides empirical evidence for institutional theory perspectives on sustainability adoption in hospitality (25). Mega-events create coercive pressures (regulations, requirements), normative pressures (industry standards, certifications), and mimetic pressures (best practice adoption) that accelerate sustainability implementation. However, the variation in outcomes across cases suggests that institutional pressures alone do not guarantee successful implementation—organizational capacity and commitment remain critical factors (26).

The study illustrates that hotels respond to institutional pressures in different ways based on their existing capabilities, resource constraints, and strategic agendas. Hotels that already have sustainability practices can leverage mega-event pressures to advance improvements, but inexperienced hotels might struggle to implement major changes within event timeframes. The finding contributes to institutional theory by demonstrating the requirement of organizational readiness for moderating institutional impacts.

Third, the study highlights the complexity of the business case for sustainability in hospitality. While eco-efficiency measures often demonstrate clear ROI, our findings support Melissen et al.'s (8) argument that comprehensive sustainability requires acceptance of initiatives without direct financial returns. This challenges simplistic “win-win” narratives and aligns with critical perspectives on corporate sustainability (38, 39).

The concept of “sustainability-enabled resilience” is a concept emerging from our research, and its implications are that hotels adopting overall sustainability strategies in the context of mega-events build adaptation capabilities that pay off well beyond the life of the event. Specifically, this contribution to hospitality resilience theory illustrates how sustainability strategies and measures can be at once ends and means of building organizational resilience.

The hospitality industry has been increasingly adopting green technologies, though significant implementation challenges remain (96). Olympic Agenda 2020 has further emphasized sustainability as a core pillar for mega-events (97).

Our findings must be interpreted in light of the well-documented intention-behavior gap in sustainable tourism (34). While 73% of tourists express preference for sustainable hotels (3), actual booking behaviors often prioritize other factors. Hotels investing in sustainability based on stated preferences may overestimate demand, particularly for measures that increase costs without enhancing guest experience.

This gap manifests differently during mega-events. Event attendees may be less price-sensitive than typical tourists, potentially reducing barriers to choosing sustainable options. However, convenience factors (proximity to venues, availability during peak periods) may override sustainability considerations. Hotels must therefore frame sustainability initiatives not solely as responding to guest demands but as fulfilling broader stakeholder expectations and regulatory requirements.

The evidence is directed to the business case of sustainability in hotel operations of mega-events as complicated. Since eco-efficiency efforts are likely to reflect clear ROI by way of reduced operating costs, our findings substantiate Melissen et al.'s (8) argument that serious sustainability in hotels must embrace actions without manifest monetary benefits. This challenges naive “win-win” arguments and agrees with critics' arguments regarding corporate sustainability (38, 39). Our analysis reveals that mega-event sustainability initiatives encompass more than eco-efficiency measures. Environmental sustainability includes investments in renewable energy infrastructure, biodiversity conservation, and circular economy practices—many without immediate financial returns. Social sustainability dimensions include: These findings reiterate the importance of explicitly anchoring sustainability legacies within hotel operations—highlighting how hotel-specific constraints and incentives shape long-term outcomes.

• Workforce development: Training programs, fair labor practices, career advancement opportunities
• Community engagement: Local hiring, supplier diversity, cultural preservation
• Accessibility: Infrastructure improvements benefiting persons with disabilities
• Human rights: Particularly salient in Qatar 2022, where labor conditions overshadowed environmental initiatives

These broader sustainability dimensions align with evolving frameworks such as the UN Sustainable Development Goals and emerging regulatory requirements like the EU Corporate Sustainability Reporting Directive (CSRD). Hotels must increasingly account for Scope 3 emissions (including guest travel) and demonstrate progress on social indicators, challenging narrow focus on operational eco-efficiency (90).

The variation in outcomes across cases underscores the importance of governance structures and accountability mechanisms. London 2012's independent oversight body provided credibility and enabled adaptive management. Conversely, Qatar 2022's disputed carbon-neutral claims illustrate how inadequate transparency can undermine legitimacy, regardless of actual achievements.

This finding supports calls for standardized reporting frameworks and third-party verification in event sustainability (91). The adoption of ISO 20121 (sustainable event management) and alignment with GRI standards represents progress, but enforcement remains inconsistent. Future events must balance ambitious targets with credible measurement and reporting to avoid greenwashing accusations.

Our analysis reveals that sustainability legacies are neither uniformly positive nor represent clear “win-win” scenarios. Several factors complicate outcomes:

1. Temporal dynamics: Short-term gains may not translate to long-term benefits without continued investment
2. Distributional effects: Benefits and costs unevenly distributed across stakeholder groups
3. Opportunity costs: Resources directed to event sustainability might achieve greater impact elsewhere
4. Attribution challenges: Difficulty isolating event impacts from broader industry trends

These complexities challenge deterministic views of mega-event legacies and support more nuanced, context-sensitive approaches to evaluation (92, 93).

For event organizers and policymakers:

• Integrate sustainability planning from bid stage, not as add-on
• Establish independent oversight with transparent reporting
• Set realistic targets based on implementation capacity
• Create mechanisms for post-event legacy management

For hotel operators:

• View mega-events as catalysts for accelerating existing sustainability plans
• Invest in initiatives with co-benefits (e.g., efficiency + guest experience)
• Prepare for evolving regulations beyond voluntary standards
• Document and share learnings for industry advancement

For certification bodies:

• Harmonize standards to reduce confusion and costs
• Strengthen verification processes to ensure credibility
• Develop event-specific modules recognizing unique demands
• Track long-term outcomes beyond initial certification

## Conclusion

This study examined how mega-events influence sustainability practices in the hotel sector, revealing complex patterns of adoption, implementation, and legacy. While events can catalyze significant improvements in environmental and social performance, outcomes depend critically on governance structures, stakeholder commitment, and post-event management. The findings challenge simplistic narratives of universal “win-win” outcomes, instead revealing trade-offs between different sustainability dimensions and stakeholder interests.

The research contributes to theoretical understanding of legacy creation, institutional pressures in sustainability adoption, and the evolving business case for comprehensive sustainability beyond eco-efficiency. Practical implications include the need for integrated planning, transparent accountability mechanisms, and recognition that meaningful sustainability requires accepting some initiatives without direct financial returns.

Future research should employ longitudinal designs to track legacy evolution, examine differential impacts across stakeholder groups, and investigate how emerging regulations (e.g., CSRD, mandatory climate disclosures) influence event and hotel sustainability practices. As the climate crisis intensifies and social inequalities persist, the imperative for genuinely sustainable mega-events—and the hospitality infrastructure supporting them—will only grow stronger.
